# Developmental delays and dental caries in low-income preschoolers in the USA: a pilot cross-sectional study and preliminary explanatory model

**DOI:** 10.1186/1472-6831-13-53

**Published:** 2013-10-12

**Authors:** Donald L Chi, Katharine C Rossitch, Elizabeth M Beeles

**Affiliations:** 1 University of Washington, Box 357475, Seattle 98195, WA, USA

## Abstract

**Background:**

Anecdotal evidence suggests that low-income preschoolers with developmental delays are at increased risk for dental caries and poor oral health, but there are no published studies based on empirical data. The purpose of this pilot study was two-fold: to examine the relationship between developmental delays and dental caries in low-income preschoolers and to present a preliminary explanatory model on the determinants of caries for enrollees in Head Start, a U.S. school readiness program for low-income preschool-aged children.

**Methods:**

Data were collected on preschoolers ages 3–5 years at two Head Start centers in Washington, USA (N = 115). The predictor variable was developmental delay status (no/yes). The outcome variable was the prevalence of decayed, missing, and filled surfaces (dmfs) on primary teeth. We used multiple variable Poisson regression models to test the hypothesis that within a population of low-income preschoolers, those with developmental delays would have increased dmfs prevalence than those without developmental delays.

**Results:**

Seventeen percent of preschoolers had a developmental delay and 51.3% of preschoolers had ≥1 dmfs. Preschoolers with developmental delays had a dmfs prevalence ratio that was 1.26 times as high as preschoolers without developmental delays (95% CI: 1.01, 1.58; P < .04). Other factors associated with increased dmfs prevalence ratios included: not having a dental home (P = .01); low caregiver education (P < .001); and living in a non-fluoridated community (P < .001).

**Conclusions:**

Our pilot data suggest that developmental delays among low-income preschoolers are associated with increased primary tooth dmfs. Additional research is needed to further examine this relationship. Future interventions and policies should focus on caries prevention strategies within settings like Head Start classrooms that serve low-income preschool-aged children with additional targeted home- and community-based interventions for those with developmental delays.

## Background

Dental caries is the most common disease in children [1]. Recent epidemiologic data from the U.S. National Health and Nutrition Examination Survey (NHANES) suggest that dental caries prevalence among preschool children ages 2–5 years increased by 15.1% (from 24.2% in 1988–1994 to 27.9% in 1999–2004) [2]. Furthermore, from 1999–2004, 47.8% of preschoolers from low-income households experienced caries and 35% had untreated caries (compared to 11.4% and 6% of preschoolers from higher income households, respectively) [3,4]. These data underscore the association between poverty and poor oral health [5-7] in preschoolers and raise public health concerns, particularly in regards to the U.S. Healthy People 2020 objectives that call for reductions in the percentage of preschoolers with dental caries experience and untreated dental decay to 33.3% and 23.8%, respectively [8].

Poor oral health is associated with school absenteeism, learning problems, and pain [9] as well as systemic disease, hospitalization, and in rare cases death [10]. Oral diseases during early childhood are likely to have health consequences over the life course [11], which highlights the importance of caries prevention strategies, particularly for low-income preschoolers.

The U.S. Head Start program was promulgated in 1965 to address disparities in school readiness for low-income preschoolers. Head Start emphasizes cognitive and social development as well as health promotion and nutrition [12]. The program focuses on low-income preschoolers and was founded on the premise that improving nutritional intake and health outcomes can help to reduce disparities in school readiness [13]. At the start of each school year, all Head Start enrollees are evaluated by an education specialist to identify special health care needs, which are defined as “deafness, speech or language impairments, visual impairments including blindness, serious emotional disturbance, orthopedic impairments, autism, traumatic brain injury” or developmental delays [12]. For Head Start children identified with a developmental delay, Lead Education Agencies are responsible for providing tailored Individualized Education Programs (IEPs) [14]. IEPs are written documents that describe the child’s specific delay, skills that need to be developed, services the school will provide, and where the services will take place. In 2009, there were over 900,000 Head Start enrollees in the U.S. [15] and 12% of enrollees had an IEP [16].

In regards to dental care, nearly 85% of Head Start enrollees received preventive dental care and 88% had a dental examination in the 2010–2011 program year [16]. These data suggest that Head Start has reduced some of the documented barriers to dental care for low-income preschoolers [17,18]. However, dental caries prevalence among Head Start enrollees remains high, ranging from 38% in Connecticut to 86% in Florida [13-23]. A 2005 prospective study reported that providing dental care coordination services to the caregivers of Head Start enrollees improved dental use for children but did not improve oral health status [24]. Collectively, these findings suggest that interventions focusing solely on increasing dental care utilization are insufficient in preventing dental disease in low-income preschoolers served by the Head Start program.

Targeted interventions, such as school-based sealant programs, have the potential to improve the oral health of children at greatest risk for poor oral health [17]. Anecdotal evidence suggests that preschoolers with developmental delays are at increased risk for dental caries, but there are no published studies to support this hypothesis. The current pilot study was guided by an adapted version of Patrick’s sociocultural oral health disparities model [25], which posits that the determinants of dental caries in vulnerable children are multifactorial. We tested two hypotheses: 1) low-income preschoolers with developmental delays have greater dental caries prevalence (measured by dmfs) than those without developmental delays; and 2) other factors are associated with dental caries in low-income preschoolers.

## Methods

Study Design, Participants, and Location. This was a cross-sectional pilot study based on secondary data. The study focused on preschoolers ages 3–5 years in two Head Start classrooms in Washington, USA (N = 115). Both classrooms were located in Kittitas County, a rural county in eastern Washington. Over 92% of Kittitas County is White compared to 82.0% for Washington state [26]. The median household income was $42,769 and 22.3% of individuals were below the Federal Poverty Level ($58,890 and 12.5%, respectively, for Washington state) [26]. We received human subjects approval to conduct this study from the University of Washington Institutional Review Board.

### Conceptual model

A sociocultural model on oral health disparities presented by Patrick and colleagues was adapted to generate a preliminary conceptual model [25]. This model posits that social and cultural factors from multiple levels influence oral health outcomes for vulnerable populations, including low-income preschool-aged children. The original model posits that these multilevel factors interrelate directly and indirectly to produce oral health disparities within vulnerable populations. Our parsimonious model conceptualized covariates as direct correlates of dental caries and each covariate was classified into one of four domains:

***Ascribed factors*** (immutable individual-level demographic characteristics: age, sex, race);

***Proximal factors*** (modifiable individual-level behavioral characteristics: communication difficulties; dental home);

***Immediate factors*** (family-level interpersonal factors: primary caregiver’s education; primary caregiver’s employment status; family structure; home health environment);

***Distal factors*** (system-level environment: community water fluoridation).

### Data sources

There were two data sources: Head Start enrollment and health history forms. The enrollment form contained demographic information about the child (e.g., age, sex, race, Individual Education Program [IEP] participation) and the primary caregiver (e.g., education, employment, household structure). The health history form contained information on whether the child had difficulties communicating, had a dental home (or a place to take their child for dental care), lived in a smoke-free household, and lived in a fluoridated community. All data were from the 2010–2011 Head Start school year.

### Outcome measure

The outcome measure was the number of decayed, missing, or filled surfaces (dmfs) on primary teeth, a composite measure of dental caries and treatment experience. We used the National Institute of Dental and Craniofacial Research (NIDCR) Early Childhood Caries Collaborating Centers (EC4) criteria [27], which are based on the World Health Organization (WHO) methods [28]. The WHO methods define decay on pit and fissure or smooth surfaces as “an unmistakable cavity, undermined enamel, or a detectable softened floor or wall” [28]. To account for trauma and natural exfoliation, a surface was classified as missing only if the tooth was missing because of caries. Surfaces restored with amalgam, composite, glass ionomer, or stainless steel crowns were classified as filled. Sealed surfaces were classified as sound. Consistent with EC4 criteria, if there was uncertainty about the status of a tooth surface, the surface was classified into the more conservative category. Surface-level caries data were collected by a single trained and calibrated pediatric dentist. Five-percent of the study population was randomly selected for a second caries exam to allow for an assessment of intrarater reliability. The Kappa statistic was used to assess for intrarater consistency in the caries data. The intrarater reliability for the caries exam data was found to be Kappa = 0.69 (95% CI: 0.61, 0.76; P < .001), which indicates substantial agreement.

### Predictor variable

The main predictor variable was the child’s developmental delay status, defined as whether the child had an Individualized Education Program (IEP) (no/yes). While there are limitations associated with using IEP as a proxy for developmental delays (e.g., under-identification of disabilities), nearly 20% of enrollees in our study had an IEP, which approximates the 12% of Head Start children nationally with an IEP [16] and the 33% prevalence estimate of delay from a previous study [29].

### Model covariates

There were 10 model covariates hypothesized as correlates of dmfs or as confounders of the relationship between developmental delays and dmfs. These covariates were classified into four domains (see Conceptual Model subsection).

There were three ascribed factors that were modeled as confounders: age (3/4/5 years) [30]; sex (female/male) [30]; and race (non-White/White) [31].

There were two binary proximal covariates (no/yes): communication difficulty and dental home. Communication difficulty, assessed by a Head Start teacher, was measured using the communication subsection of the Ages and Stages Questionnaire, 2nd Edition (ASQ). The ASQ is a validated age-specific screener used to assess multiple developmental domains such as communication, motor, problem solving, and personal-social skills [32]. Children scoring greater than 38 points, 39 points, or 31 points on the communication subsection of the 36-, 48-, and 60-month ASQ, respectively, were classified as having no communication difficulties. Remaining children were classified as having communication difficulties. Dental home was assessed by asking the caregiver whether they needed assistance finding a dentist (no/yes) and measured whether the child had a place to go for regular preventive care and restorative dental when needed.

There were four caregiver-reported immediate covariates: caregiver education (less than high school; high school; greater than high school) [30]; caregiver employment status (unemployed; in school/training; employed) [33]; family structure (defined as whether the child lived in a single parent or two parent household) [34]; and whether the child lived in a smoke-free home (no/yes) [35], a proxy for the home health environment.

There was one caregiver-reported distal covariate: whether the child lived in a community with fluoridated water (no/yes) [36].

### Statistical analyses

We did not calculate statistical power based on previous work cautioning against power calculations for retrospective studies [37]. After generating descriptive statistics, we used the Pearson chi-square test to assess the relationships between model covariates and the main predictor variable (developmental delay status) (α = 0.05). Because the outcome was not normally distributed, we used the Wilcoxon-Mann-Whitney U test to compare median dmfs rates across model covariates. A multiple variable Poisson regression model was generated to test our hypothesis that the dmfs prevalence rate would be higher in children with developmental delays (GENLIN function with log link). Poisson regression results were presented as regression parameters (i.e., beta coefficients) with standard errors and prevalence ratios. There was no evidence of collinearity between model covariates (e.g., developmental delays and communication difficulties) and all covariates were included in the final regression model. We used PASW Statistics version 18.0 for Windows (Chicago, IL).

## Results

### Descriptive statistics

There were 115 preschoolers in our study and 17.4% were identified with a developmental delay (Table 1). Thirteen percent of preschoolers had a communication problem and 91.3% had a dental home. Caregiver education level was evenly distributed across the three categories and 27.8% of caregivers were unemployed. Nearly 90% of preschoolers lived in a smoke-free home and 64% lived in communities with fluoridated water. Significantly larger proportions of preschoolers with a developmental delay were male compared to preschoolers without a developmental delay (75% and 44.2%, respectively; P = .012).

**Table 1:** Pearson Chi-Square test results for bivariate relationships between developmental delay status (Predictor Variable) and model covariates for Head Start children (N = 115)

**Variable**	**Total study population**	**Children with no developmental delay**	**Children with developmental delay**	**P-value**
	**(N = 115)**	**(n = 95)**	**(n = 20)**	
	**n (%)**	**n (%)**	**n (%)**	
**Ascribed factors**				
Age (years)				P = .46
3	22 (19.1)	2 (10.0)	20 (21.1)	
4	51 (44.3)	9 (45.0)	42 (44.2)	
5	42 (36.5)	9 (45.0)	33 (34.7)	
Sex				P = .01
Male	57 (49.6)	42 (44.2)	15 (75.0)	
Female	58 (50.4)	53 (55.8)	5 (25.0)	
Race				P = .11
White	56 (48.7)	43 (45.3)	13 (85.0)	
Non-White	59 (51.3)	52 (54.7)	7 (35.0)	
**Proximal factors**				
Communication difficulties				P = .15
No	96 (87.3)	83 (89.2)	13 (76.5)	
Yes	14 (12.7)	10 (10.8)	4 (23.5)	
Dental home				P = .52
No	10 (8.7)	9 (9.5)	1 (5.0)	
Yes	105 (91.3)	86 (90.5)	19 (95.00	
**Immediate factors**				
Caregiver education level				P = .64
Less than high school	37 (32.2)	30 (31.6)	7 (35.0)	
High school	39 (33.9)	34 (35.8)	5 (25.0)	
More than high school	39 (33.9)	31 (32.6)	8 (40.0)	
Caregiver employment				P = .86
Unemployed	32 (27.8)	26 (27.4)	6 (30.0)	
In school or training	14 (12.2)	11 (11.6)	3 (15.0)	
Employed	69 (60.0)	58 (61.1)	11 (55.0)	
Family structure				P = .62
Single parent	46 (40.0)	39 (41.1)	7 (35.0)	
Two parents	69 (60.0)	56 (58.9)	13 (65.0)	
Child lives in a smoke-free home				P = .18
No	13 (11.4)	9 (9.6)	4 (20.0)	
Yes	101 (88.6)	85 (90.4)	16 (80.0)	
**Macro factor**				
Lives in a community with fluoridated water				P = .68
No	40 (36.0)	32 (35.2)	8 (40.0)	
Yes	71 (64.0)	59 (64.8)	12 (60.0)	

Nearly 49% of children had zero dmfs (data not shown). The mean dmfs was 5.8 (standard deviation: 11.2 dmfs; median: 1.0 dmfs; maximum: 65 dmfs). The mean number of decayed, filled, and missing surfaces was 1.3, 4.0, and 0.5, respectively. There were no significant differences in the median dmfs rates across all model covariates (Table 2).

**Table 2:** **Wilcoxon-Mann-Whitney U test results for bivariate relationship between *decayed, missing, or filled tooth surfaces (dmfs) *** **(Outcome Measure) and model covariates for Head Start children (N = 115)**

**Variable**	**Median *dmfs***	**P-value**
	**(N = 115)**	
**Main predictor variable**		
Developmental delay		P = .92
No	1.0	
Yes	0.5	
**Ascribed factors**		
Age (years)		P = .27
3	0.0	
4	1.0	
5	2.0	
Sex		P = .30
Male	0.0	
Female	2.0	
Race		P = .24
White	0.0	
Non-White	2.0	
**Proximal factors**		
Communication Difficulties		P = .47
No	0.5	
Yes	1.5	
Dental home		P = .30
No	4.0	
Yes	0.0	
**Immediate factors**		
Caregiver education level		P = .42
Less than high school	0.0	
High school	2.0	
More than high school	0.0	
Caregiver employment		P = .66
Unemployed	2.0	
In school or training	2.0	
Employed	0.0	
Family structure		P = .73
Single parent	0.5	
Two parents	1.0	
Child lives in a smoke-free home		P = .32
No	1.0	
Yes	0.0	
**Macro factor**		
Lives in a community with fluoridated water		P = .32
No	1.0	
Yes	0.0	

### Regression models

The covariate-adjusted Poisson regression model indicated that developmental delays were significantly associated with dmfs (Table 3). Preschoolers with developmental delays had a dmfs prevalence ratio that was 1.26 times as high as children without developmental delays (95% CI: 1.01, 1.58; P < .04). Of the 10 remaining model covariates, six covariates across all four model domains were significantly associated with dmfs (age, communication difficulties, dental home, caregiver education level, caregiver unemployment, and living in a community with fluoridated water). Older preschoolers as well as preschoolers with communication difficulties (ascribed and proximal factors, respectively), those with caregivers who finished high school or less (an immediate factor), and children with an unemployed caregiver (also an immediate facto) had increased dmfs prevalence ratio. Preschoolers with a dental home (a proximal factor) and those living in communities with fluoridated water (a macro factor) had significantly decreased dmfs prevalence ratios.

**Table 3:** Final multiple variable poisson regression model on dental caries prevalence for Head Start children (N = 115)

**Variable**	**Parameter estimate (B)**	**Standard error**	**95% CI**	**Prevalence ratio (PR)**	**95% CI**	**P-value**
**Main predictor variable**						
Developmental delay						P = .04
No	ref	-	-	-	-	
Yes	0.23	0.12	0.01, 0.46	1.26	1.01, 1.58	
**Ascribed factors**						
Age (years)						
3	ref	-	-	-	-	-
4	0.49	0.15	0.20, 0.79	1.64	1.22, 2.20	P = .001
5	1.44	0.15	1.13, 1.73	4.21	3.08, 5.64	P < .001
Sex						P = .41
Male	ref	-	-	-	-	
Female	−0.07	0.09	−0.25, 0.10	0.93	0.78, 1.11	
Race						P = .42
White	ref	-	-	-	-	
Non-White	−0.09	0.11	−0.32, 0.13	0.91	0.73, 1.14	
**Proximal factors**						
Communication difficulties						P < .001
No	ref	-	-	-	-	
Yes	0.91	0.15	0.62, 1.19	2.47	1.85, 3.30	
Dental home						P = .01
No	ref	-	-	-	-	
Yes	−0.49	0.19	−0.87, -0.11	0.61	0.42, 0.89	
**Immediate factors**						
Caregiver education level						
Less than high school	0.95	0.17	0.62, 1.13	2.58	1.85, 3.09	P < .001
High school	0.86	0.13	0.61, 1.11	2.36	1.84, 3.03	P < .001
More than high school	ref	-	-	-	-	-
Caregiver employment		-				
Unemployed	ref		-	-	-	-
In school or training	0.32	0.15	0.04, 0.61	1.38	1.04, 1.83	P = .03
Employed	−0.83	0.10	−1.02, -0.64	0.44	0.36, 0.53	P < .001
Family Structure						P = .51
Single parent	ref	-	-	-	-	
Two parents	0.06	0.10	−0.13, 0.26	1.07	0.88, 1.29	
Child’s lives in a smoke-free home						P = .55
No	ref	-	-	-	-	
Yes	−0.07	0.12	−0.31, 0.17	0.93	0.73, 1.18	
**Macro factor**						
Lives in a community with fluoridated water						P < .001
No	ref	-	-	-	-	
Yes	−0.99	0.10	−1.17, -0.80	0.37	0.31, 0.45	

## Discussion

This is the first published study to examine the relationship between developmental delays and dental caries in low-income preschool-aged children. We tested two hypotheses within a population of preschoolers in the Head Start program. The first hypothesis was that the dmfs prevalence ratio would be higher for Head Start preschoolers with developmental delays than for Head Start preschoolers without. Our findings support this hypothesis. There are no studies to which we can directly compare our results, but there are three potential explanations. First, preschoolers with developmental delays may not cooperate with home care behaviors such as toothbrushing, which leads to plaque accumulation and limited exposure to topical fluorides. Second, preschoolers with developmental delays may be exposed more frequently to fermentable carbohydrates (e.g., medications, sugar sweetened beverages, sweets). Third, caregivers of preschoolers with disabilities may experience higher levels of caregiver stress [38], which could exacerbate the preceding factors that contribute to poor oral health. These findings suggest that low-income preschool-aged children with developmental delays are a vulnerable subgroup among low-income preschoolers.

The second hypothesis was that other factors would be related to dmfs. Six model covariates were significantly associated with caries: age, communication difficulties, not having a dental home, lower caregiver education, unemployment, and living in a community with non-fluoridated water. Previous studies support our findings regarding age [27]. In terms of the significant proximal factors (communication difficulties and dental home) there are no studies to which we can directly compare our findings. However, two studies suggest a relationship between child temperament and caries [39,40]. In our study, there was low correlation between communication difficulties and developmental delays, suggesting that these measures capture different aspects of child behaviors. Additional research is needed to identify the mechanisms by which communication difficulties can lead to increased caries. Furthermore, in regards to the dental home variable, a preliminary study reported that young children have less tooth decay when their mothers have a dental home [41]. Our findings are the first to suggest an association between children having a dental home and lower caries experience rates. While dental homes are considered to be important by parents and dentists [42,43], there are few relevant studies to which we can compare our findings. Children with a dental home may have caregivers with good oral health behaviors (e.g., prevention-oriented dental care use, healthy eating, regular home oral hygiene). We recognize the limitations associated with our operationalization of the dental home, which is an area of dental research that requires additional attention. Future research should continue to test different operationalizations of the dental home concept, evaluate clinical outcomes associated with dental homes, and identify the specific features of dental homes that lead to good oral health.

There were also two significant immediate factors: caregiver education level and employment status. There is extensive literature on the oral health effects of low caregiver education, which is associated with low health literacy, negative oral health-related behaviors, and social disadvantage [44-47]. In terms of employment effects, compared to preschoolers with an unemployed caregiver, preschoolers with an employed caregiver had significantly fewer caries whereas preschoolers with a caregiver in school had significantly greater caries. A potential explanation is that employed caregivers may have greater flexibility to take time off from work to take their child to the dentist. Caregivers in school may rely on relatives for caretaking responsibilities, leaving them fewer opportunities to oversee enforcement of toothbrushing and healthy eating. Our findings conflict with a recent study from Australia, which reported no relationship between employment and caries in 20-month old children but reported a significant interaction between employment and family structure [33]. Broadly, there is growing recognition that addressing the social determinants of pediatric health such as caregiver education and employment, has the potential to improve various health outcomes, including oral health [48]. Our findings underscore the importance of identifying the specific factors associated with employment that could promote child health outcomes such as time-flexible work policies [49] and examining how children’s oral health is influenced by interactions between employment and family-level factors. Dental health professionals also have a responsibility to partner with the health policy and public health communities to help craft social and economic policies that seek to improve the upstream determinants of health as a way to achieve oral health equity in vulnerable populations.

The only macro factor in our model (living in a community with fluoridated water) was significantly associated with fewer caries. There are numerous studies that support the benefits associated community water fluoridation [36,50,51]. Because segments of the population are concerned with the safety of or oppose community water fluoridation [52], there is a need for continued research on the behavioral and social determinants of opposition to water fluoridation. Policies and interventions must be developed to ensure that health professionals have the resources to inform patients and the public about the importance of community water fluoridation.

Also of interest are the two immediate factors (family structure and living in a smoke-free home) and the two ascribed factors (sex and race) that failed to reach statistical significance in our regression model. Our finding that family structure was not associated with caries is inconsistent with a previous study reporting that children from one-parent families had significantly higher caries rates than those from two-parent families [34]. Our results are also inconsistent with previous studies that link caregiver smoking and caries [35,53-55]. One potential explanation is social desirability bias regarding reliable reporting of smoking status [56]. We would not expect differences in dental caries prevalence by sex, as demonstrated in our model, but a previous study found that female infants had greater odds of developing severe caries [31]. Furthermore, in our model, race failed to reach statistical significance, which is inconsistent with previous findings [57]. A possible explanation is low variance in regards to non-White children in our study population, most of whom were of Hispanic or Latino descent. Future research should continue to examine how features associated with households, home health environment, and race/ethnicity are related to dental disease in young children.

**Figure 1:**
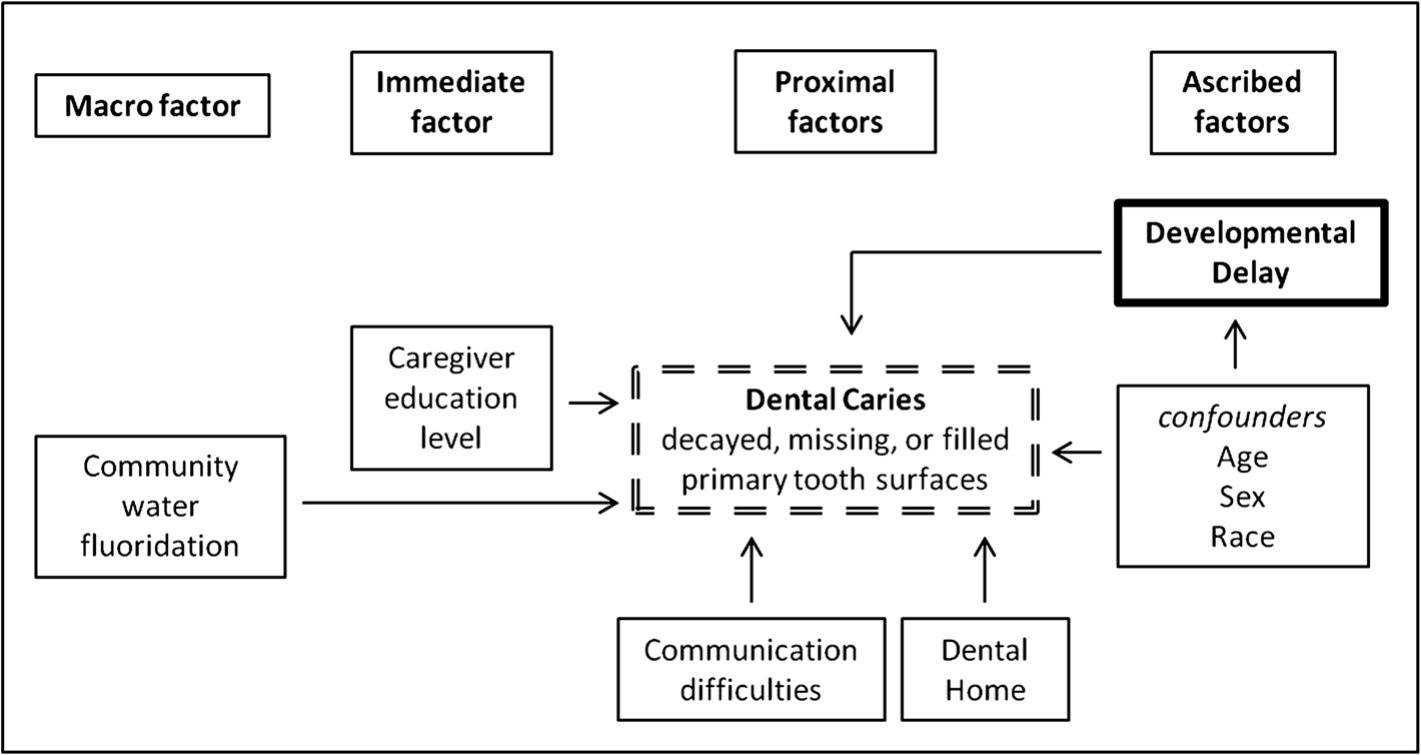
**Sample figure title.** Preliminary conceptual model on the multilevel factors associated with dental caries experience in Head Start children in the U.S.

Collectively, our findings support a preliminary conceptual model on dental caries for low-income preschoolers enrolled in the Head Start program (Figure 1). There are two features of this model. The first is that the correlates of primary tooth dmfs are found at multiple levels. Our model suggests that reducing dental caries in low-income preschool-aged populations requires complex interventions that reach beyond single-level approaches such as ensuring dental homes or community water fluoridation [58]. Limited financial and human resources coupled with persisting caries prevalence rates among vulnerable populations indicate the need for innovative strategies that address the multilevel determinants of poor oral health. This is related to the second feature of the model – the mutability of model covariates. Some of the model features (e.g., developmental delays, caregiver education level, employment) are immutable in the short-term, which represents opportunities to implement targeted interventions and policies. For instance, if children with developmental delays are at greater risk for dmfs, as demonstrated in our study, targeted interventions should focus on these preschoolers rather than all Head Start enrollees. Other model features (e.g., dental home, community water fluoridation) are mutable and may serve as active ingredients in a targeted intervention. For example, an intervention focusing on children with developmental delays could include case managers who work with caregivers and community dentists to ensure that the child is seen regularly by a dentist for checkups and treatment as necessary and behavioral interventions that reinforce use of fluoridated water, regular toothbrushing with fluoride toothpaste, and healthy diet. Additional research is needed to refine and validate our preliminary dental caries model so that appropriate interventions and policies can be developed and tested.

Increases in dental caries prevalence in preschool-aged children in the U.S. have renewed interest in population-based strategies to prevent and manage dental disease in young children [59]. Intensive multilevel interventions implemented within Head Start classrooms coupled with community- and home-based strategies for the highest risk children may be needed to achieve meaningful health improvements [60]. Head Start programs should implement and test preventive strategies within classrooms (e.g., twice daily toothbrushing with fluoridated toothpaste, diet control, iodine and fluoride varnish applications) [61,62]. A recent study conducted within Head Start classrooms suggests that fluoride-xylitol toothpastes are not more efficacious than fluoride-only toothpastes [63]. Research is needed to evaluate the efficacy and acceptability of additional preventive strategies that could be implemented within Head Start classrooms such as toothbrushing with higher concentration fluoride products and distributing snacks containing therapeutic levels of xylitol [64,65]. Head Start teachers and caregivers will require training about dental disease prevention and how to properly implement these strategies [66-68]. Beyond the classroom setting, there are promising opportunities to implement caregiver-, household-, and community-level interventions that target Head Start enrollees with developmental delays [69,70]. These efforts will require rigorous evaluation so that interventions can be modified as needed and disseminated to other settings.

This study has a number of strengths including adaptation of a conceptual framework that guided all stages of the study, assessment of intrarater reliability for the clinical caries data, and blinding of the caries examiner. However, as with all studies, there were limitations. The first is that our conceptual model is likely to be incomplete. Because of data limitations, we were unable to include all cultural, social, and environmental factors from Patrick’s model (e.g., cultural attitudes toward oral health, norms, social capital, social disadvantage, area-level poverty). Future work could investigate additional cultural and biopsychosocial factors related to dental caries in young children [71]. Second, the data were cross-sectional and there is no assumption of causality. Longitudinal studies are needed to better understand how risk factors influence oral health outcomes overtime. Third, the study focused on two Head Start classrooms in a rural county, which limits external generalizability of our study findings. There is a need to conduct larger studies that include Head Start classrooms from a variety of geographic settings.

## Conclusions

Based on the results of this pilot study, we draw two conclusions. There was a significant positive association between developmental delays and dmfs prevalence in low-income preschool-aged children served by Head Start. In addition, factors such as having a dental home and living in a community with fluoridated water were associated with significantly lower dmfs prevalence ratios. Additional studies are needed to further examine the relationship between developmental delays and primary tooth caries in preschoolers, the mechanisms underlying this relationship, and multilevel strategies to reduce oral health disparities in vulnerable preschool-aged children.

## Competing interests

The authors declared that they have no competing interests.

## Authors’ contributions

DLC conceptualized the study, led the data collection, oversaw data analyses, and led in writing the initial and final manuscript. KCR helped to collect and manage data, helped with the data analyses, and assisted with writing the initial and final manuscript. EMB helped to college and manage data and helped write the initial and final manuscript. All authors read and approved the final manuscript.

## Pre-publication history

The pre-publication history for this paper can be accessed here:

http://www.biomedcentral.com/1472-6831/13/53/prepub
